# Deep Learning Based Antenna Selection for MIMO SDR System †

**DOI:** 10.3390/s20236987

**Published:** 2020-12-07

**Authors:** Shida Zhong, Haogang Feng, Peichang Zhang, Jiajun Xu, Huancong Luo, Jihong Zhang, Tao Yuan, Lei Huang

**Affiliations:** 1 College of Electronics and Information Engineering, Shenzhen University, Nanhai Avenue 3688, Shenzhen 518060, China; shida.zhong@szu.edu.cn (S.Z.); fenghaogang@email.szu.edu.cn (H.F.); xujiajun2017@email.szu.edu.cn (J.X.); 2170269134@email.szu.edu.cn (H.L.); zhangjh@szu.edu.cn (J.Z.); lhuang@szu.edu.cn (L.H.); 2 Guangdong Provincial Mobile Terminal Microwave and Millimeter Wave Antenna Engineering Research Center, College of Electronics and Information Engineering, Shenzhen University, Shenzhen 518060, China; yuantao@szu.edu.cn

**Keywords:** antenna selection, deep learning, multiple-input multiple-output (MIMO), software defined radio (SDR), deep neural network (DNN)

## Abstract

In this paper, we propose and implement a novel framework of deep learning based antenna selection (DLBAS)-aided multiple-input–multiple-output (MIMO) software defined radio (SDR) system. The system is constructed with the following three steps: (1) a MIMO SDR communication platform is first constructed, which is capable of achieving uplink communication from users to the base station via time division duplex (TDD); (2) we use the deep neural network (DNN) from our previous work to construct a deep learning decision server to assist the MIMO SDR platform for making intelligent decision for antenna selection, which transforms the optimization-driven decision making method into a data-driven decision making method; and (3) we set up the deep learning decision server as a multithreading server to improve the resource utilization ratio. To evaluate the performance of the DLBAS-aided MIMO SDR system, a norm-based antenna selection (NBAS) scheme is selected for comparison. The results show that the proposed DLBAS scheme performed equally to the NBAS scheme in real-time and out-performed the MIMO system without AS with up to 53% improvement on average channel capacity gain.

## 1. Introduction

Massive multiple-input–multiple-output (MIMO), which is one of the most promising techniques in the 5G as well as the coming 6G wireless communication [1,2,3], shows its superiority in terms of Rayleigh-fading resistance, high channel capacity, considerable spectral efficiency, etc. In recent year, there have been more and more theoretical studies on massive MIMO systems (e.g., [4,5,6]). However, multiple radio frequency (RF) chains, which are linked with the same number of antennas, are equipped in the conventional MIMO systems, which leads to high hardware costs and power consumption. In particular, there are usually hundreds of RF chains equipped in massive MU-MIMO systems, while the number of RF chains in practical systems is often limited. As a remedy, antenna selection (AS) techniques are capable of retaining MIMO advantages, while achieving low system complexity and reducing hardware costs. Specifically, norm-based AS (NBAS) and capacity-based AS (CBAS) are two common types of AS schemes, and, compared with CBAS, NBAS has lower computational complexity, which makes NBAS more suitable for implementing in communication systems [7]. Moreover, time division duplex (TDD) and many other technologies can be used to optimize the performance of AS [8,9].

At the same time, in 5G wireless communication, there will be a surge in the amount of communication data. How to make the best use of these information to achieve intelligent communications will be a novel research topic. In recent years, more and more attention has been paid to artificial intelligent (AI)-aided communications, which has adopted machine learning (ML) or deep learning (DL) methods to solve the mathematical problems in wireless communication systems [10,11,12,13,14,15,16,17,18,19,20], due to the fact that ML is capable of transferring the conventional mathematical optimization problems into data-driven problems for achieving lower online real-time computational complexity. More specifically, He [10] exploited the potential benefits of ML in the transmit antenna selection (TxAS)-aided MIMO system. Compared to the conventional TAS scheme, the scheme proposed in [10] is capable of achieving almost the same bit error ratio (BER) with relatively small overhead. Joung [11] was the first to employ multi-class classification algorithms in a MIMO system with AS, which interpreted the AS for MIMO communications as multi-class classification learning. The results verified the feasible complexity and performance of ML-based AS, which provided insights for the use of various ML algorithms for AS. Liao [12] adopted DL to achieve channel state information (CSI) feedback in massive MIMO system, which solved the problem of high complexity and low feedback accuracy. Additionally, Cai [13] proposed the LeNet model for receiving AS schemes, which is a single-label multi-class classification CNN model. The proposed method uses convolutional structure to extract the rich features from the channel matrices, and numerical experimental results show that the LeNet model-based AS outperformed the state-of-the-art baselines. Meanwhile, the authors of [14,15,16,17,18,19] investigated the ML-based problems related to the physical layer of MIMO. O’Shea [14] developed an end-to-end learning MIMO communication system based on unsupervised deep learning to build an autoencoder to estimate channel state and get the CSI matrix. Ma [15] proposed an end-to-end deep neural network to mimic the pilot signals and channel estimator that are acquired by data-driven deep learning in the wideband Massive MIMO systems. This novel way of using deep learning approach to jointly design pilot signals and channel estimator shows superior performance over other compressive sensing approaches. Elbir [16] formulated antenna selection and hybrid beamformer design as a classification/prediction problem for convolutional neural networks and Huang [17] provided a hybrid precoding scheme based on deep leaning. The DL can also work to replace nontrivial algorithm. Alrabeiah [18] proposed a novel concept of channel mapping in space and frequency and used deep neural networks to efficiently learn this channel mapping algorithm. Moreover, in vehicle-to-infrastructure 5G mmWave MIMO communication scenarios, Klautau [19] presented and used a deep learning based methodology for channel data generation, which combines a vehicle traffic simulator with a ray-tracing simulator. Finally, An [20] investigated the CNN-based AS-aided massive MIMO system, which uses the simulated CSI data to train CNN model for generating AS decision. The results show that the CNN-based AS was capable of achieving the optimal AS performance when considering antenna number is equal to 32.

However, few of the studies on DL-based AS methods mentioned above measured the realistic communication performance due to the hardware limitations. As a remedy, research for the next generation communication can be accelerated by the software defined radio (SDR), which can avoid the hardware limitation [21,22,23]. The authors of [22,23] used the SDR platform to implement AS-aided MIMO communication system. By using the NI SDR MIMO-OFDM system consisting of FPGAs, Zhang [22] developed the prototype of NBAS-aided MIMO system based on an efficient NBAS algorithm, which is the first hardware system that provides a near-realistic MIMO communication system to run with NBAS algorithm. Based on a efficient zero-forcing beamforming (ZFBF), a user-oriented smart TxAS scheme was firstly implemented in SDR MIMO communication platform by Zhong [23], which could carry out AS according to various qualities of service (QoS) requirements of different users. These cases have enlightening significance for us to apply DL in SDR to solve problems in communication systems.

To the best of our knowledge, most studies on AI communications only stay at the level of theoretical research, and few of theoretical investigations are capable of achieving hardware implementation. In this paper, we propose and implement a novel DL-based antenna selection (DLBAS)-aided MIMO SDR communication system. More specifically, we separate an arithmetic unit from the MIMO SDR communication platform and build an AI decision server which uses the DNN model proposed from our previous work [24], aggregating a well-trained ML/DL model. The AI decision server applies the multithreading mechanism, which is capable of accelerating the response speed, and adopts TCP for data interaction with MIMO SDR platform, which ensures reliable data delivery.

The rest of this paper is organized as follows. Section 2 proposes the DLBAS-aided MIMO SDR communication system. In Section 3, the performance of DLBAS-aided MIMO SDR communication system is analyzed. The conclusions are given in Section 4.

## 2. The Implementation of DLBAS-Aided MIMO SDR System

**Figure 1:**
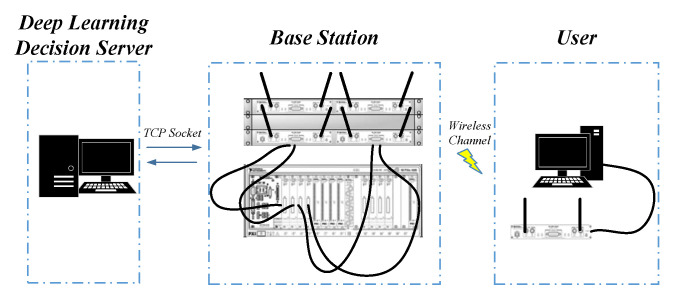
The system structure of DLBAS-aided MIMO SDR system.

In this paper, we propose a novel structure of DLBAS-aided MIMO SDR system, and its block diagram is depicted in Figure 1. The base station (BS) and the user parts in Figure 1 are used the basic structure of AS-aided MIMO system from [22,23], which are built by 8×2 AS-aided MIMO system based on NI USRP-RIO SDR platform comprising an eight-antenna BS and two single-antenna users. However, the BS in Figure 1 is modified to work with the deep learning decision server. In the downlink, after the channel estimation in the BS, the channel state information (CSI) is uploaded to the deep learning decision server, which is running on a PC to make intelligent selection for AS.

**Figure 2:**
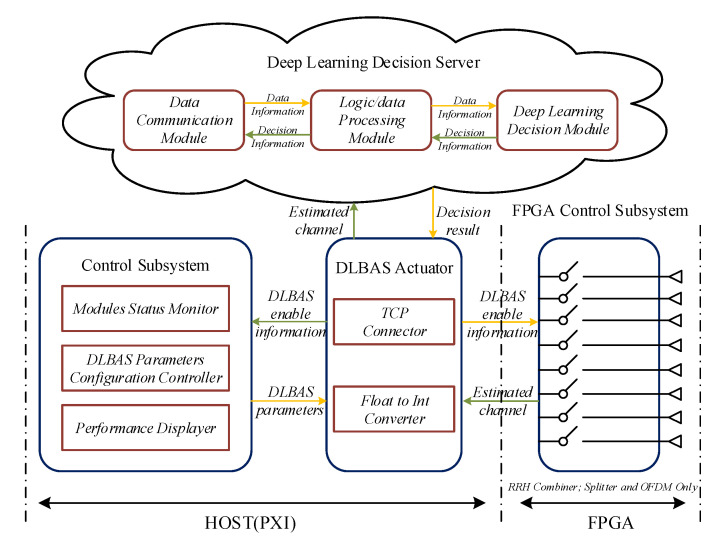
The structure of DLBAS-aided MIMO SDR system.

The structure of DLBAS-aided MIMO SDR system is shown in Figure 2. The Control Subsystem and DLBAS Actuator are running on the BS HOST. Specifically, the Control Subsystem, which consists of Modules Status Monitor, DLBAS Parameters Configuration Controller, and Performance Displayer, is used to input system parameter and display the system performance. More specifically, DLBAS Parameters Configuration Controller firstly receives the input settings from users, i.e, the number of selected antennas, the internet protocol (IP) address, and the port of Deep Learning Decision Server, which would be transmitted to the DLBAS Actuator. Moreover, during the system functioning, the Modules Status Monitor can record the working status of each module of the system, such as the status of USRP-RIOs and their antennas, and then the recorded information will be displayed by the Performance Displayer, which is helpful to check whether the various modules of the system are working properly. According to these information, DLBAS Actuator connects the DL decision server at the period of system initialization. During the system functioning, the channel state will be estimated in the BS by the channel simulation model [22], and the DLBAS Actuator uploads the estimated CSI towards DL decision server, which is introduced in Section 2.1, and receives the intelligent decision result from DL Decision Server via TCP Connector. Note that the type of estimated CSI is float, and the Float to Int Convertor is to transform the float value into integer value by IEEE 754 standard.

The FPGA Control Subsystem is running on the FPGA of each USRP-RIO. The DLBAS Actuator transmits the AS enable signals, which are updated according to the decision result received from DL decision server, to the FPGA Control Subsystem via panel (FP) transmission, which is the module used to control FPGA variables in HOST module in LabVIEW. Then, each FPGA Control Subsystem can control the data links of their USRP-RIO based on the received enable signals. The method for data link control is to regulate the data output in first-input-first-output (FIFO). More specifically, in each USRP-RIO, the data links of selected antennas stay connected, while those of unselected antennas are cut off. Data in disconnected antenna data links need to keep forward to prevent blocking the FIFO.

A deep learning algorithm is adopted to build a predictive model functioning in the Deep Learning Decision Server to make intelligent decision. The following subsections describe the details of implementing the DLBAS scheme.

### 2.1. The Structure of Deep Learning Decision Server

Based on the structure of DLBAS-aided MIMO SDR system mentioned above, we design a DL decision server to assist the SDR platform for making intelligent decision, implying that the data-driven decision making method takes place in the optimization-driven decision making method. The top part of Figure 2 depicts the block diagram of DL decision server. In Figure 2, it is obvious that the DL decision server consists of three parts: Data Communication module, Logic/Data Processing module, and Deep Learning Decision module. The detail information of each module is as follows.

#### 2.1.1. Deep Learning Decision Module

During the system functioning, a huge amount of data on historical scenarios may have been acquired and stored at the HOST. Many optimal or near-optimal solutions are exploited to deal with these data as the computing capability of the computer becoming stronger. By extracting the feature in these historical scenarios data, the DL decision module can be forwarded to guide BS how to make decision for antenna selection in MIMO communication system.

**Figure 3:**
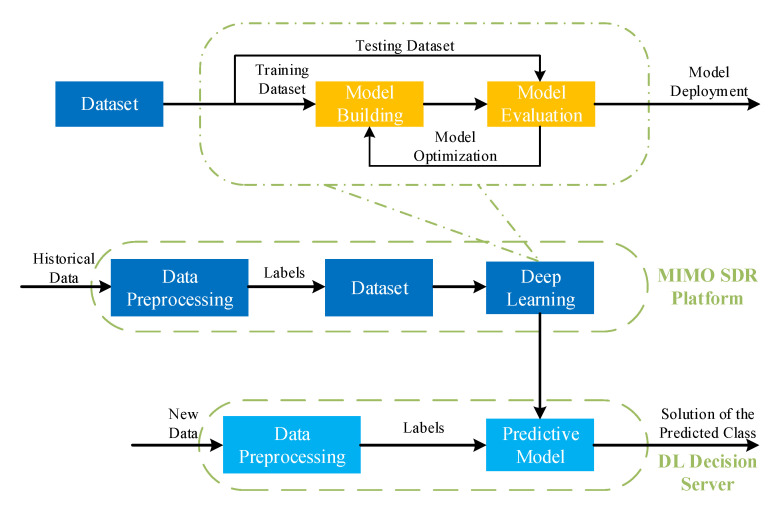
The process of DL decision module design.

The process of DL decision module is shown in Figure 3. At the MIMO SDR platform, many historical data are acquired. To construct the dataset, at first, as data preprocessing, feature selection is carried out to identify and remove some irrelevant attributes of historical data. Through feature selection, some key attributes are selected and presented as a feature vector. This will improve the quality of the dataset and enable the machine learning process to function more effectively. Further, a label is needed for each feature vector because a supervised learning algorithm in machine learning is adopted. More specifically, optimal or near-optimal solutions will be adopted to find the label of each feature vector. Therefore, the feature vector with the same solutions belongs to one class and as many data as possible of each class will be collected. To build the machine learning model, all samples in the dataset are split randomly into training and testing datasets. Normally, 70–90% of the samples are assigned into the training dataset, and the other samples are assigned into the testing dataset. The training set is used to build a machine learning model and the testing dataset is used to evaluate the model. Through model optimization, a predictive model can be built which will be used to make decision for new data. More specifically, with the aid of machine learning model, a high performance solution of future real-time scenarios can be searched offline and can give a decision in time. All the real-time scenarios are transformed into a mathematical multi-class classification problem and the predictive model will predict one class for one new feature vector. The classifier can be described as

(1) $$\begin{array}{c} l=Classifier(F_{T}), \end{array}$$

where $F_{T}$ is the new feature vector extracted from the new data, and *l* is the output class index showing that the new data belongs to the *l*th class.

Although computing resources are consumed to build a predictive model, the computing work can be carried out offline. Moreover, the new feature vector will be collected and forwarded to update the dataset, which is very important for tracing the evolution of real scenarios, including user behaviors and wireless propagation environment. The dataset can be used to develop the machine learning model and all this work can also be accomplished during the off-peak time.

#### 2.1.2. Logic/Data Processing Module

The Logic/Data Processing Module is used as a bridge between the Data Communication Module and Deep Learning Decision Module. First, the Logic/Data Processing Module is capable of undoing the header of information to extract the payload in the received information. Then, the payload is sent as input of the Deep Learning Decision Module for intelligent decision output.

**Figure 4:**
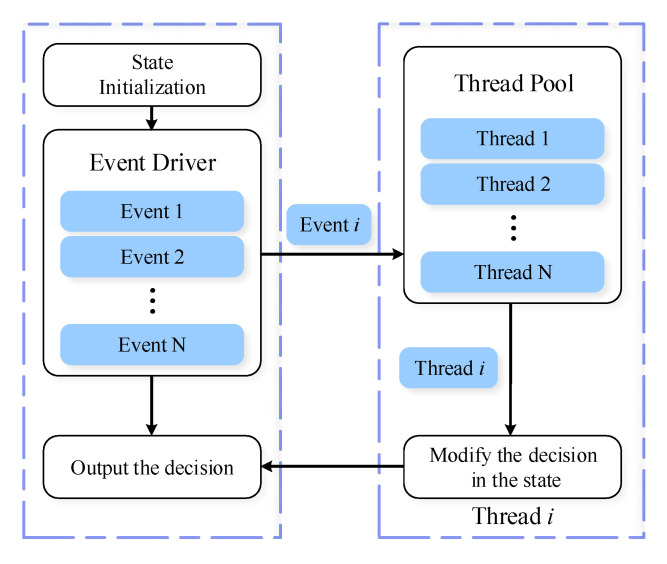
The process of multithreading logic.

Second, to improve the resource utilization ratio of DL decision server, we set up the DL decision server as a multithreading server, which is asynchronous event-driven. Therefore, the Logic/Data Processing Module needs to cope with the communication logic between different threads, as shown in Figure 4. More specifically, the Logic/Data Processing Module manages a container, which saves the state of event drivers and the decision result. Firstly, the module initializes its state. Then, the main thread in the Logic/Data Processing Module selects one assistance thread corresponding to the active event driver. The selected thread may generate new DL decision, modify the state of the decision result in the container, and kill itself at last. Finally, the main thread reads the decision saved in the container and transmits the result to Data Communication Module.

#### 2.1.3. Data Communication Module

Data Communication Module is responsible for connecting DL decision server with the MIMO SDR platform via transmission control protocol (TCP).

**Figure 5:**
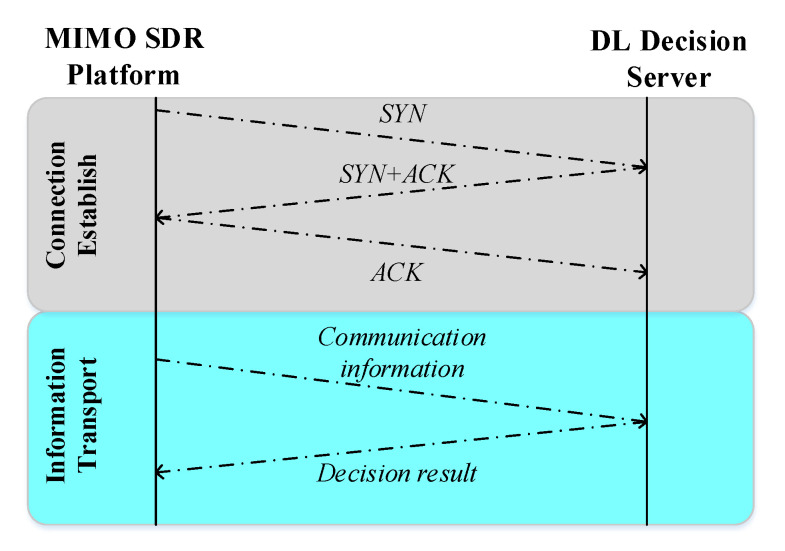
The process of TCP connection between the MIMO SDR platform and DL decision server.

The process of TCP connection between the MIMO SDR platform and DL decision server is depicted in Figure 5. Owing to TCP’s advantages of connection-oriented property and ordered transmission, the DL decision server can achieve reliable data communication with less overhead. In the period of system initialization, the DL decision server listens for MIMO SDR platform access, which is a client/server model. Once MIMO SDR platform sends its request for access, it would establish a connection with DL decision server through TCP three-way handshake protocol. Then, the MIMO SDR platform can transmit some communication data towards Data Communication module in DL decision server, as shown in Figure 2, while the decision result at DL decision server would be sent to the MIMO SDR platform via Data Communication module.

### 2.2. The Design of Dataset and Neural Network for Antenna Selection

We considered different machine learning methods for AS, such as deep neural network (DNN), K-nearest neighbors (KNN), and eXtreme Gradient Boosting (XGBoost) algorithm, and the results demonstrate that DNN has better performance on AS [24]. Therefore, we adopt DNN to construct the classification model for MIMO antenna selection. The implementation process of DNN for AS in the SDR MIMO platform is introduced in the following subsections.

#### 2.2.1. The Generation of Dataset

To construct neural network classification model, the dataset is firstly obtained, which consists of CSI samples and labels from MIMO SDR system. Then system trains the DNN using the obtained dataset. Due to the limitation of our MIMO SDR hardware setup, the current measured dataset only supports a maximum of eight antennas in the base station with two single-antenna users. In this way, when new CSI comes in, by using the trained DNN classification model, we obtain the optimal antenna subset which can achieve better channel capacity.

Let C denote the field of complex numbers. Firstly, we obtain the estimated CSI $H\in C^{N\times M}$ from MIMO SDR platform, which can be expressed as

(2) $$\begin{array}{c} H=\left[\begin{array}{cccc} h_{11} & h_{12} & \cdots & h_{1M}\\ h_{21} & h_{22} & \cdots & h_{2M}\\ \vdots & \vdots & \ddots & \vdots \\ h_{N1} & h_{N2} & \cdots & h_{NM} \end{array}\right], \end{array}$$

where $h_{ij}$ denotes the (*i*, *j*)th complex value element of H and *N* and *M* stand for the number of receive antennas and transmit antennas, respectively. Then, by exploiting the estimated CSI as sample data, we use the optimal or near-optimal result of norm-based antenna selection (NBAS) algorithm described in [22] as the corresponding label. In DNN-based systems, the training dataset is required to be real-valued numbers, while the MIMO channels are complex-valued. In this case, the steps of dataset acquisition are summarized in Algorithm 1:
**Algorithm 1** Dataset acquisition process  1:  **repeat**  2:  Convert the estimated CSI matrix H to a real-value vector $h\in R^{1\times NM}$ as
(3)$$h=[|h_{11}|,|h_{12}|,\cdots ,|h_{1M}|,|h_{21}|,|h_{22}|,\cdots ,|h_{2M}|,\cdots ,|h_{NM}|].$$  3:  Obtain the optimal antenna subset by NBAS. The antenna subset is replaced by the label     $l\in [l_{1},l_{2},\cdots ,l_{s}]$, where s=NNs and $N_{s}$ denotes the number of selected receive antennas.  4:  Convert the label *l* to one-hot vector **z**.  5:  **until** All samples are generated

#### 2.2.2. The Design of Neural Network

**Figure 6:**
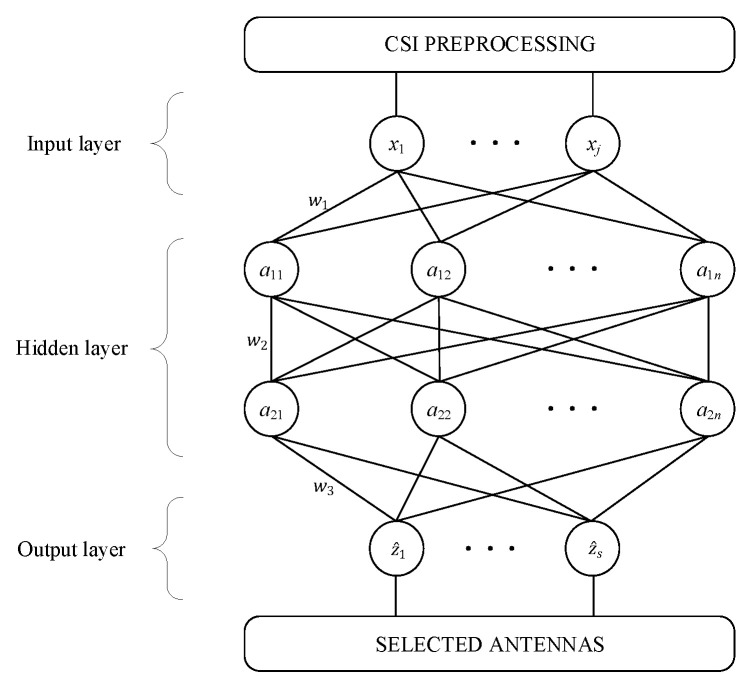
The structure of self-designed neural network for antenna selection.

In the DNN module, we opt for using the fully connected neural network to select the optimal antenna subset, of which the corresponding DNN model refers to and improves the method in [24], and it is shown in Figure 6.

It can be seen that the self-designed DNN model consists of an input layer, two hidden layers, and an output layer [25]. The input layer is an input vector $h_{j}=\left[h_{1},\cdots ,h_{NM}\right]$ with 1×NM representing the processed real-valued CSI according to Equation (3). Each hidden layer has 500 nodes and more hidden layers will make the model more complicated. $W_{i}$ is the weighting matrix and its dimension jointly depends on the number of nodes of (*i* − 1)th layer and *i*th layer. $a_{ij}$ corresponds to the *i*th layer output vector, and we usually employ rectified linear unit (ReLU), of which the form is

(4) $$\begin{array}{c} f(c)=\max(0,c), \end{array}$$

as the nonlinear activation function. The output vector of the *j*th sample in the output layer can be expressed as

(5) $$\begin{array}{c} {\hat{z}}_{j}=\left[{\hat{z}}_{1},\cdots ,{\hat{z}}_{s}\right]. \end{array}$$

For example, the output vector of the *l*th class of antenna subset is

(6) $$\begin{array}{c} {\hat{z}}_{j}=\left[\underset{l-1}{\underset{︸}{0,\cdots ,0}},1,\underset{s-l}{\underset{︸}{0,\cdots ,0}}\right]. \end{array}$$

In the phase of neural network training, real-valued matrix X and corresponding one-hot label matrix Z are input to the network. For each training sample input, we have a *s*-dimension vector $\hat{z}$ as the output. With using $K_{1}$ training samples, stochastic gradient descent algorithm will be employed to optimize the cross-entropy loss function between true label z and predicted label $\hat{z}$. The corresponding loss function is formulated as

(7) $$\begin{array}{c} L\left(z,\hat{z}\right)=\frac{1}{K_{1}}\sum_{i=1}^{K_{1}}(z_{i}\log\left({\hat{z}}_{i})\right)+\left(1-z_{i}\right)\log\left(1-{\hat{z}}_{i}\right). \end{array}$$

Additionally, to further optimize the DNN model used in the proposed AS-aided MIMO system, $L_{2}$ regularization technique is utilized in the loss function to avoid overfitting and the moving average model is invoked to make the final model more robust.

#### 2.2.3. The Implementation of Deep Learning Decision Server

As mentioned in Section 2.1, the DL decision server is a multithreading server based on asynchronous event-driven, implying that a multithreading model can achieve the mechanism of event-driven in DL decision server. As mentioned in Section 2.1.2, the Logic/Data Processing module in DL decision server manages a container. This container, as illustrated in Table 1, includes two control signals as event drivers, “IFCALCULATE” and “ISRESULT”, which are both initialized as false, and a memory to save the decision result. In the DL decision server, the main thread always connects to SDR platform via TCP and the control signals in the container are used to control the execution of assistance thread to update the decision result in the memory of the container.

**Figure 7:**
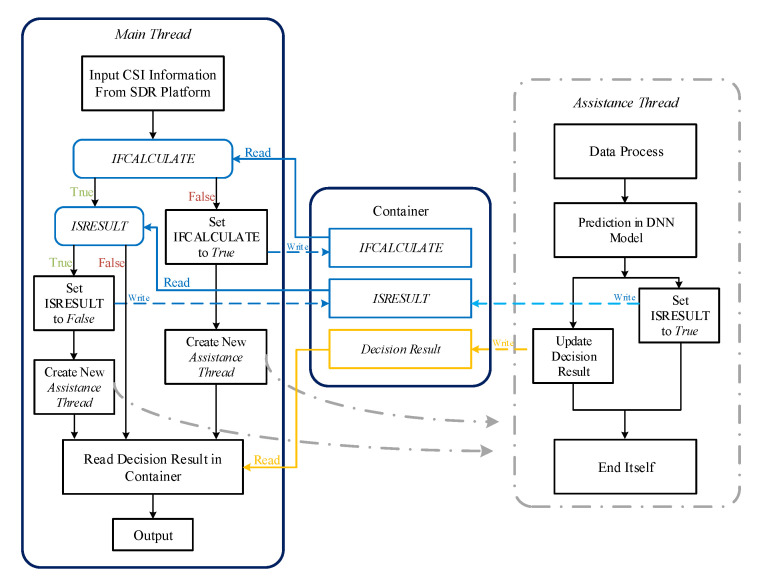
The logic of the DL decision server in DLBAS-aided MIMO SDR system.

The logic of the DL decision server in our implemented system is shown in Figure 7. More specifically, when the connection between the DL decision server and SDR platform has been established, the main thread would firstly check the state of the control signal “IFCALCULATE” in the container. If “IFCALCULATE” is False, the DL decision server would create one assistance thread to input CSI from SDR platform into DNN model and update the state of decision result and control signal “ISRESULT” in the container. The DL decision server continues to check the state of the control signal “ISRESULT” when “IFCALCULATE” is True. If “ISRESULT” is False, the DL decision server directly outputs the decision result in the container. The DL decision server outputs the updated decision result and sets “ISRESULT” to False to create an assistance thread when “ISRESULT” is True.

## 3. Experiment Results and Analysis

In this section, our experiments are based on the constructed DLBAS-aided MIMO-OFDM SDR communication system shown in Figure 2 and described in Section 2, where an eight-antenna BS and a single-antenna user are employed. The MIMO SDR system equipped with *N* receive antennas, as well as required Ns selected receive antennas, is denoted by (*N*/Ns). To illustrate the channel capacity performance of DLBAS-aided MIMO systems, a NBAS-aided MIMO system and a no AS MIMO system, as reported in [22], are considered in this section for comparison. Moreover, the prediction precision performance of our designed DNN is also analyzed in our experiments.

### 3.1. The Performance of DNN Model

**Figure 8:**
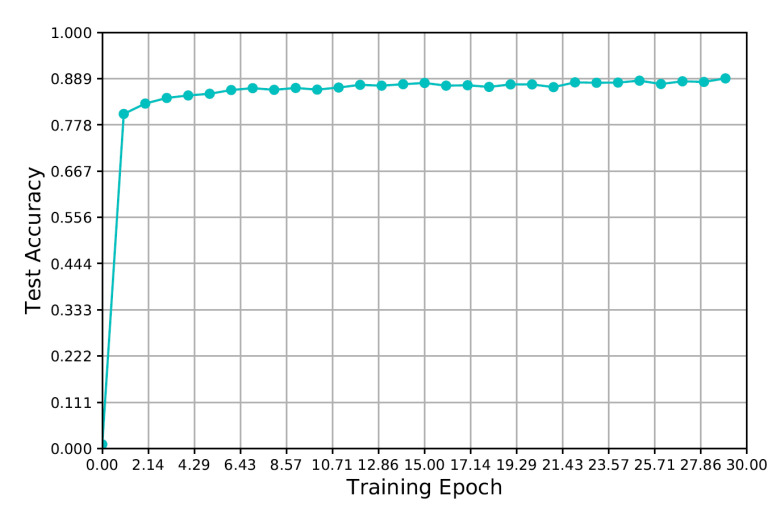
The trends of test accuracy of the designed DNN model.

The test accuracy during the training of the designed DNN model for antenna selection is shown in Figure 8. It can be seen that the test performances keep growing in Figure 8 with the increasing of training epoch and it will be remain stable at about 0.89 in later training periods. Moreover, as shown in Figure 8, the test accuracy is about to reach fit at Epoch 2, which means that the data-fitting speed of the designed DNN is very fast.

The computational complexity of our DNN model is compared with other AS schemes in [24]. For further implementation in Massive MIMO communication system, we consider the prediction time complexity of the network [26]; the computational complexity of the DNN model may be presented as

(8) $$\begin{array}{c} O(M\cdot N\cdot s)\propto O(M\cdot N) \end{array}$$

### 3.2. Results of DLBAS

**Figure 9:**
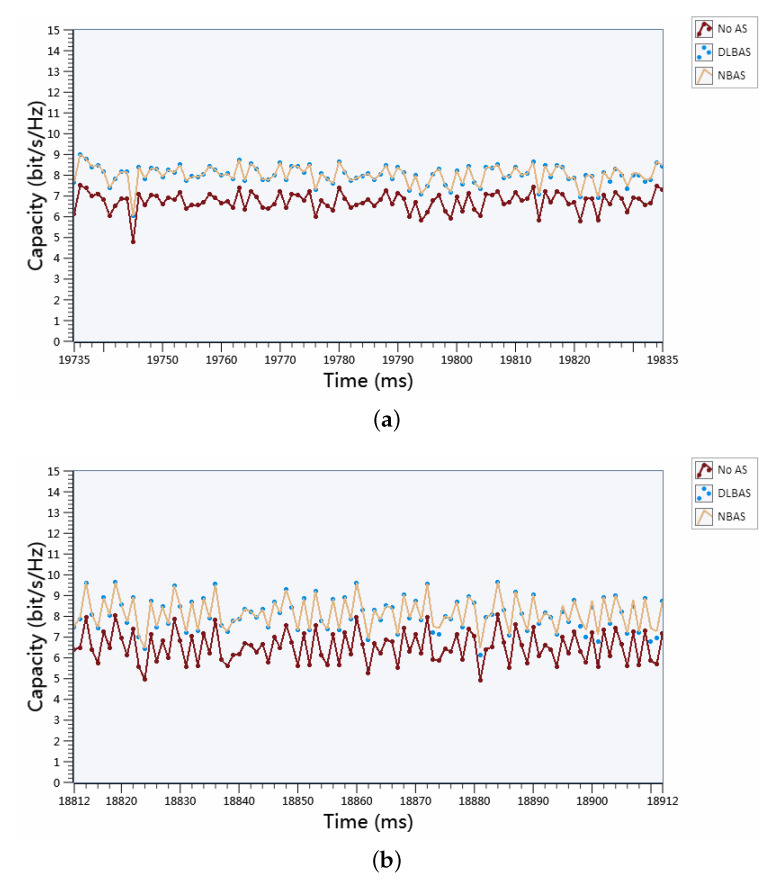
Real-time channel capacities comparison: (**a**) (8/2) DLBAS-aided MIMO SDR system, (8/2) NBAS-aided MIMO SDR system, and (2/2) no AS-aided MIMO SDR system; and (**b**) (8/4) DLBAS-aided MIMO SDR system, (8/4) NBAS-aided MIMO SDR system, and (4/4) no AS-aided MIMO SDR system.

In different SNR environments with perfect CSI, the comparison of real-time channel capacities of three kinds of algorithm, DLBAS, NBAS, and no AS, during the system functioning is depicted in Figure 9. Figure 9a shows the capacities of these three algorithms when N=8, $N_{s}=2$, while Figure 9b displays that of the three algorithms when N=8, $N_{s}=4$. In Figure 9, the proposed DLBAS algorithm approaches the NBAS algorithm and outperforms no AS, implying that the DLBAS has almost the same channel capacity performance as NBAS. More specifically, in Figure 9a, the channel capacities of (8/2) DLBAS-aided MIMO SDR system and (8/2) NBAS-aided MIMO SDR system are both 9bits/s/Hz at the 19,780th ms, while the capacity of (2/2) no AS-aided MIMO SDR system is around 7.5bit/s/Hz, which shows about 1.5bits/s/Hz performance gain. Moreover, in Figure 9b, the channel capacities of (8/4) DLBAS-aided MIMO SDR system and (8/4) NBAS-aided MIMO SDR system agree in 10bits/s/Hz at the 18,860th ms, when the capacity of (4/4) no AS-aided MIMO SDR system is around 8bits/s/Hz, which attains 2bits/s/Hz performance gain. Moreover, Figure 9 reveals that DLBAS is capable of choosing the best antenna selection choice at most of the time. Therefore, the experimental results in Figure 9a,b demonstrate that the proposed DLBAS scheme performed equally to the NBAS scheme in different SNR environment, implying the correctness of our DLBAS scheme.

**Figure 10:**
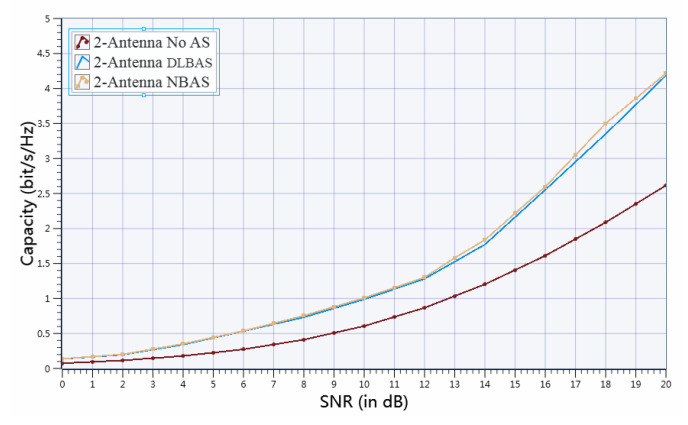
Measurement result of channel capacity comparison among (8/2) DLBAS-aided MIMO SDR system, (8/2) NBAS-aided MIMO SDR system, and (2/2) no AS-aided MIMO SDR system.

**Figure 11:**
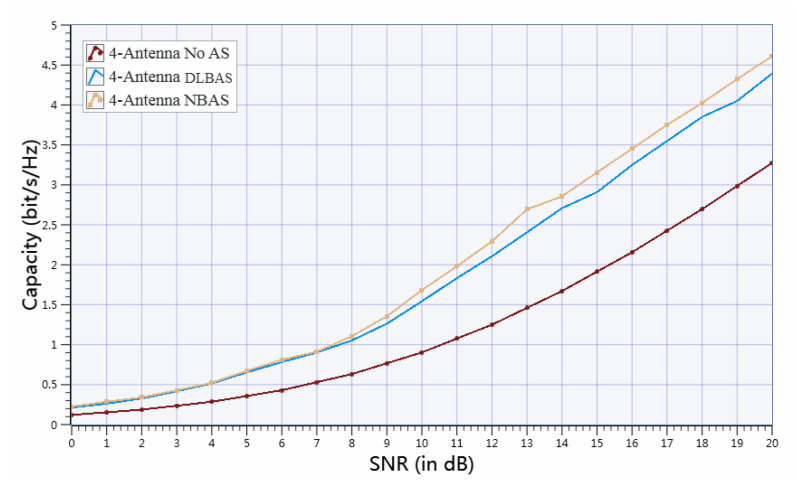
Measurement result of channel capacity comparison among (8/4) DLBAS-aided MIMO SDR system, (8/4) NBAS-aided MIMO SDR system, and (4/4) no AS-aided MIMO SDR system.

In our experiments, SNR can be adjusted by changing the direction of antennas or adding obstacles in the wireless communication environment in order to add noise to plot the measured channel capacity vs. SNR curves. The achievable capacity performances recorded for the (8/2) DLBAS-aided MIMO SDR system, (8/2) NBAS-aided MIMO SDR system, and (2/2) no AS-aided MIMO SDR system in measurement are shown in Figure 10. It is obvious that the channel capacity results of (8/2) DLBAS-aided MIMO SDR system and (8/2) NBAS-aided MIMO SDR system are better than (2/2) no AS-aided MIMO SDR system across SNR from 0 to 20 dB, and the average channel capacity gain is about 59%, as shown in Figure 10. More specifically, when SNR=20 dB, the measured capacities of (8/2) DLBAS-aided MIMO SDR system and (8/2) NBAS-aided MIMO SDR system are both around 4.2bits/s/Hz and that of (2/2) no AS-aided MIMO SDR system is 2.6bits/s/Hz, which shows 61% channel capacity gain. Moreover, Figure 11 depicts the channel capacity vs. SNR curves comparison among (8/4) DLBAS-aided MIMO SDR system, (8/4) NBAS-aided MIMO SDR system, and (4/4) no AS-aided MIMO SDR system to further prove the same trend of channel capacity gain in measurement. Specifically, the measured channel capacity results of (8/4) DLBAS-aided MIMO SDR system is better than (4/4) no AS-aided MIMO SDR system across SNR from 0 to 20 dB, and the average channel capacity gain is about 53%. Meanwhile, when SNR=10 dB, the measured capacity of (8/4) DLBAS-aided MIMO SDR system is around 1.5bits/s/Hz and that of (4/4) no AS-aided MIMO SDR system is 0.9bits/s/Hz, which shows 70% channel capacity gain.

However, compared with Figure 10, Figure 11 shows that the measured capacity from (8/4) NBAS-aided MIMO SDR system is slightly higher than (8/4) DLBAS-aided MIMO SDR system; for example, the measured capacity at SNR=16dB of (8/4) NBAS-aided MIMO SDR system in Figure 11 is about 0.2 bps/Hz higher than (8/4) DLBAS-aided MIMO SDR system in Figure 11 because the (8/4) DLBAS-aided MIMO SDR system needs more classes than (8/2) DLBAS-aided MIMO SDR system in the DNN classification model, which results in a decrease in the test accuracy. Overall, the results of the NBAS-aided MIMO SDR system and DLBAS-aided MIMO SDR system in Figure 11 are still close.

## 4. Conclusions

In this paper, a DLBAS-aided MIMO SDR system is designed and implemented. By adopting the simple yet efficient concept of DL decision server in the MIMO SDR platform, all the real-time scenarios are transformed into a mathematical multiclass classification problem. Via TCP transmission, all the AS decision work can be done online with very low latency. The experimental results verified that the DLBAS-aided MIMO SDR system is capable of achieving near-optimal capacity performance of NBAS-aided MIMO SDR platform, which shows great improvements compared to the MIMO SDR platform without AS, and the average channel capacity gain exceeds 53%. This novel implementation of DLBAS-aided MIMO SDR system will provide practical verification environment for new design and evolution of other new AS algorithms in the MIMO communication system. Our future work is to apply this implementation framework to a real massive MIMO system, which will require more USRPs to support at least 32 antennas in the base station and also more user nodes. To keep the accuracy of the antenna selection result, the DNN model shown in Figure 6 may need to add more layers and more nodes in each layer. This will require more measured datasets and increased training time of the DNN model, but, according to Equation (8), the runtime of generating AS decision will increase slightly.

## Figures and Tables

**Table 1:** The content of the container in DL decision server.

Variable Name	Type
IFCALCULATE	boolean
ISRESULT	boolean
Decision Result	integer
